# PD-L1 expression in medulloblastoma: an evaluation by subgroup

**DOI:** 10.18632/oncotarget.24951

**Published:** 2018-04-10

**Authors:** Allison M. Martin, Christopher J. Nirschl, Magda J. Polanczyk, W Robert Bell, Thomas R. Nirschl, Sarah Harris-Bookman, Jillian Phallen, Jessica Hicks, Daniel Martinez, Aleksandra Ogurtsova, Haiying Xu, Lisa M. Sullivan, Alan K. Meeker, Eric H. Raabe, Kenneth J. Cohen, Charles G. Eberhart, Peter C. Burger, Mariarita Santi, Janis M. Taube, Drew M. Pardoll, Charles G. Drake, Michael Lim

**Affiliations:** ^1^ Johns Hopkins School of Medicine, Sidney Kimmel Cancer Center, Division of Pediatric Oncology, Baltimore, MD, USA; ^2^ Johns Hopkins School of Medicine, Sidney Kimmel Cancer Center, Division of Cancer Immunology, Baltimore, MD, USA; ^3^ Department of Laboratory Medicine and Pathology, University of Minnesota Medical School, Minneapolis, MN, USA; ^4^ Johns Hopkins School of Medicine, Department of Pathobiology, Baltimore, MD, USA; ^5^ Johns Hopkins School of Medicine, Department of Ophthalmology, Baltimore, MD, USA; ^6^ Johns Hopkins School of Medicine, Sidney Kimmel Cancer Center, Division of Cancer Biology, Baltimore, MD, USA; ^7^ Johns Hopkins School of Medicine, Department of Pathology, Division of Kidney and Urologic Pathology, Baltimore, MD, USA; ^8^ Children’s Hospital of Philadelphia, Department of Pathology and Laboratory Medicine, Philadelphia, PA, USA; ^9^ Johns Hopkins School of Medicine, Department of Dermatology, Division of Dermatologic Pathology and Oral Pathology, Baltimore, MD, USA; ^10^ In Jackson, MS, USA; ^11^ Johns Hopkins School of Medicine, Department of Pathology, Division of Neuropathology, Baltimore, MD, USA; ^12^ Columbia University Medical Center, Division of Hematology/Oncology, New York, NY, USA; ^13^ Johns Hopkins School of Medicine, Department of Neurosurgery, Division of Neurosurgical Oncology, Baltimore, MD, USA

**Keywords:** medulloblastoma, PD-1, brain tumor, PD-L1, B7-H1

## Abstract

**Background:**

This study evaluated the expression of PD-L1 and markers of immune mediated resistance in human medulloblastoma (MB), the most common malignant pediatric brain tumor.

**Results:**

Overall levels of PD-L1 in human MB were low; however, some cases demonstrated robust focal expression associated with increased immune infiltrates. The case with highest PD-L1 expression was a sonic hedgehog (SHH) MB. In cell lines, SHH MB, which are low-MYC expressing, demonstrated both constitutive and inducible expression of PD-L1 while those in Group 3/4 that expressed high levels of MYC had only inducible expression. *In vitro*, IFN-γ robustly stimulated the expression of PD-L1 in all cell lines while radiation induced variable expression. Forced high MYC expression did not significantly alter PD-L1.

**Methods:**

Human MB tumor samples were evaluated for expression of PD-L1 and immune cell markers in relation to molecular subgroup assignment. PD-L1 expression was functionally analyzed under conditions of interferon gamma (IFN-γ), radiation, and MYC overexpression.

**Conclusions:**

MB expresses low levels of PD-L1 facilitating immune escape. Importantly, T_H_1 cytokine stimulation appears to be the most potent inducer of PD-L1 expression *in vitro* suggesting that an inflamed tumor microenvironment is necessary for PD-1 pathway activation in this tumor.

## INTRODUCTION

Medulloblastoma (MB) is the most common malignant brain tumor in pediatric patients affecting as many as 500 children in the United States each year [1]. Recently, this disease has been classified into four major subgroups based on molecular genetic profiling [2–4]. Individual subgroups not only have a unique molecular signature but distinct clinical features as well [5]. Tumors in the SHH subgroup are characterized by genetic alterations activating this key developmental pathway. SHH MB usually has an intermediate prognosis except in the case of those that also have somatic alterations leading to the loss of tumor suppressor *TP53*, and expression of a dominant negative mutant p53 protein. This subset of patients has a very poor prognosis within the SHH subgroup [6]. WNT subgroup tumors have alterations in the wingless/β-catenin developmental pathway. These tumors are rare, representing <10% of all MB and have an excellent prognosis. Group 3 MB usually have high-level MYC expression, and are associated with the worst overall survival. Group 4 tumors have more heterogeneous genetic abnormalities and portend an intermediate prognosis. With the identification of MB subgroups there has been great interest in the possibility of tailoring therapy by either targeting the molecular pathways directly or risk-stratifying current therapies. Several recent studies have also evaluated possible differences in the tumor immune microenvironment between these MB subgroups suggesting mechanisms to employ specific immune based treatments [7, 8]. Immunotherapy represents an attractive treatment strategy that may eventually change the current paradigm of chemoradiotherapy. It could also represent an important salvage therapy for patients with relapsed/refractory disease that has become resistant to traditional strategies.

One of the most successful and readily available immunotherapy approaches to cancer are immune checkpoint inhibitors. Thus far the immune checkpoint molecules that have shown the most activity across cancer types have been those of the programmed death (PD-1) pathway [9]. PD-1 is a molecule expressed primarily on activated effector T cells whose physiologic role is to regulate the immune response [10]. In the setting of cancer, this includes T cells activated by antigen presenting cells (APCs) in the draining lymph node that have trafficked back to the tumor microenvironment (TME) in response to tumor antigens to become tumor infiltrating lymphocytes (TILs) [11]. Upon entering the TME they will encounter a variety of immunologic stimuli from tumor cells, tumor infiltrating myeloid cells (TIMs), and stromal tissue that will affect their fate. In the case of PD-1, it reacts directly with its’ ligands, PD-L1 and PD-L2. Although the role of PD-L2 is less clearly defined both are believed to deliver inhibitory signals that dampen effector T cell response. In the case of PD-L1 a suppressive signal is sent to the effector T cell leading to anergy, exhaustion and even apoptosis [11]. Additionally, a pro-survival signal may be sent back to the tumor as a result of this receptor-ligand interaction [11]. The expression of PD-L1 on tumors may be regulated by tumor intrinsic properties leading to its’ constitutive expression, but more commonly it is upregulated in response to TME mediated stimuli, usually interferon-gamma (IFN-γ) released by effector T cells [11]. Blocking either PD-1 or PD-L1 has led to successful therapeutic anti-cancer responses in a variety of tumor types, and PD-L1 has served as a useful predictor of response to these therapies. In multiple studies of adult cancers, higher levels of PD-L1 and a more inflamed TME correlated with a stronger response to immune checkpoint inhibitor therapy with antibodies blocking either PD-1 on T cells or its primary ligand, PD-L1, expressed on tumor cells and other cells of the TME [12–16]. Therefore, due to the typically adaptive nature of PD-L1 expression, an adaptive immune response must already be taking place in order to take full advantage of this pathway. In this study, we sought to determine the pattern of PD-L1 expression in different subgroups of MB and compare this with features of the TME. Our findings suggest that there are differences in the expression of both ligands of PD-1, PD-L1 and PD-L2, intrinsic to the subgroups of MB *in vitro*. *In vivo* these differences are not readily apparent and may be masked by the presence of a suppressive TME. The possibility that MB heterogeneity extends to the immune response of this tumor will be critical to the interpretation of clinical trials utilizing PD-1 pathway inhibitors in MB.

## RESULTS

### Variation of PD-L1 expression and immune infiltrates by molecular subgroup of human medulloblastoma

**Figure 1:**
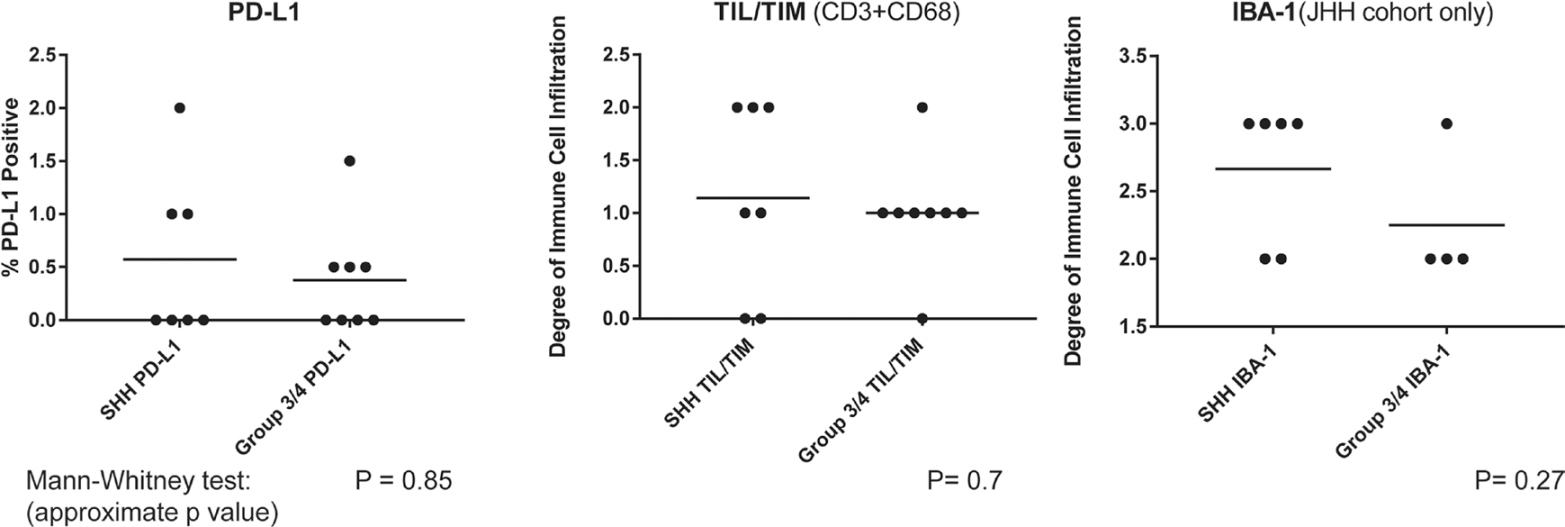
PD-L1 expression and immune infiltrates by subgroup Medulloblastoma samples were scored for tumor cell PD-L1 expression, the degree of CD3+ and CD68+ tumor infiltrating immune cells (TIL/TIM), and IBA-1+ cells and compared between SHH and Group 3/4 MB Subgroups. (IBA-1 available for JHH cohort only).

**Figure 2:**
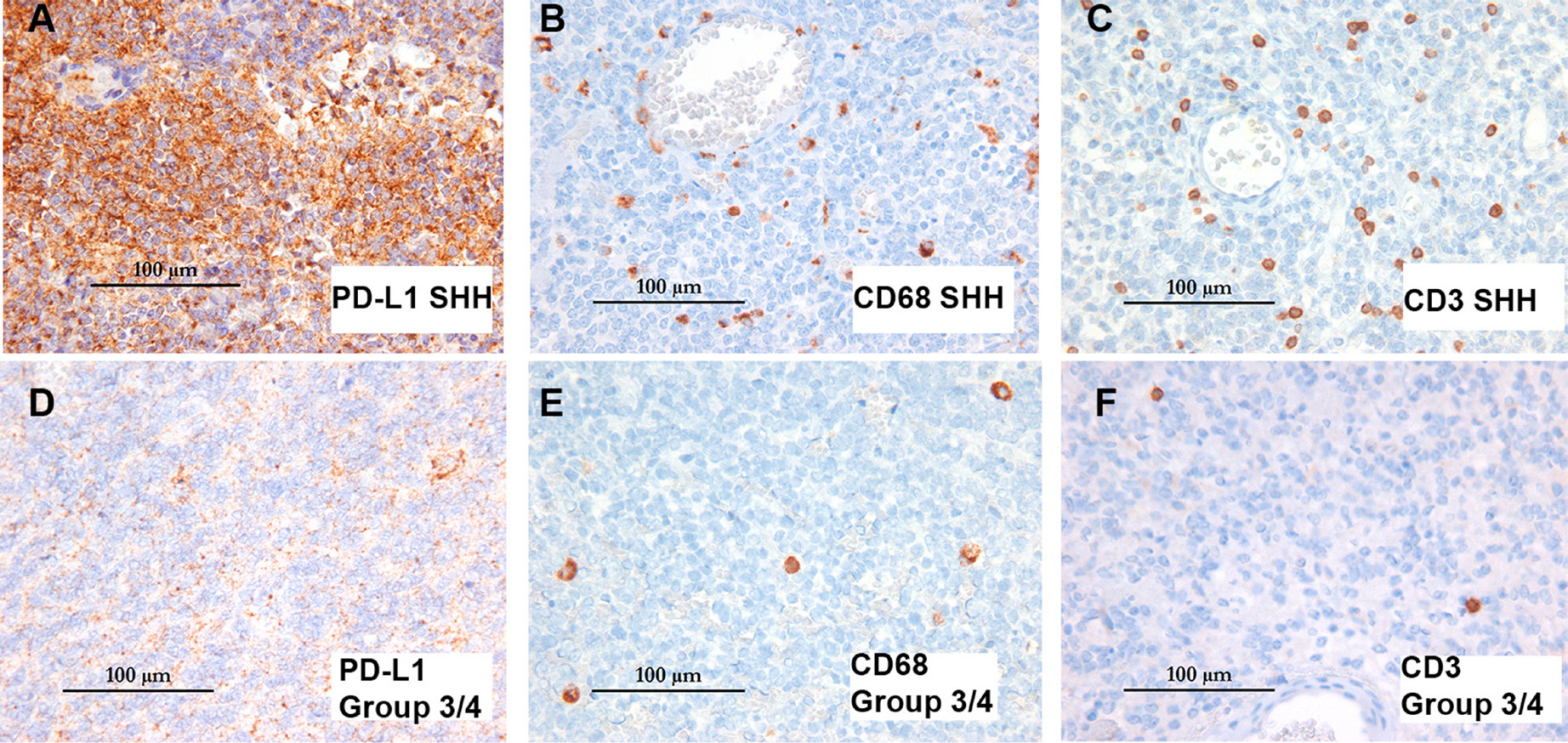
Differential expression pattern of PD-L1 and immune infiltrates in SHH vs group 3/4 MB Immunohistochemistry of PD-L1, CD3, and CD68 in two representative cases from SHH and Group 3/4 with the highest degree of PD-L1 expression in their respective subgroups. SHH case 36 (top row in **A**, **B**, **C**) had >2% PD-L1 positive staining overall and images represent an area of intense focal staining. Group 3/4 case 25 (bottom row in **D**, **E**, **F**) had 1–2% PD-L1 positive staining overall but did not demonstrate any single area of intense focal staining as seen in Case 36. Isotype antibody staining for these cases is available in Supplementary Figure 1. All images 400× original magnification.

**Figure 3:**
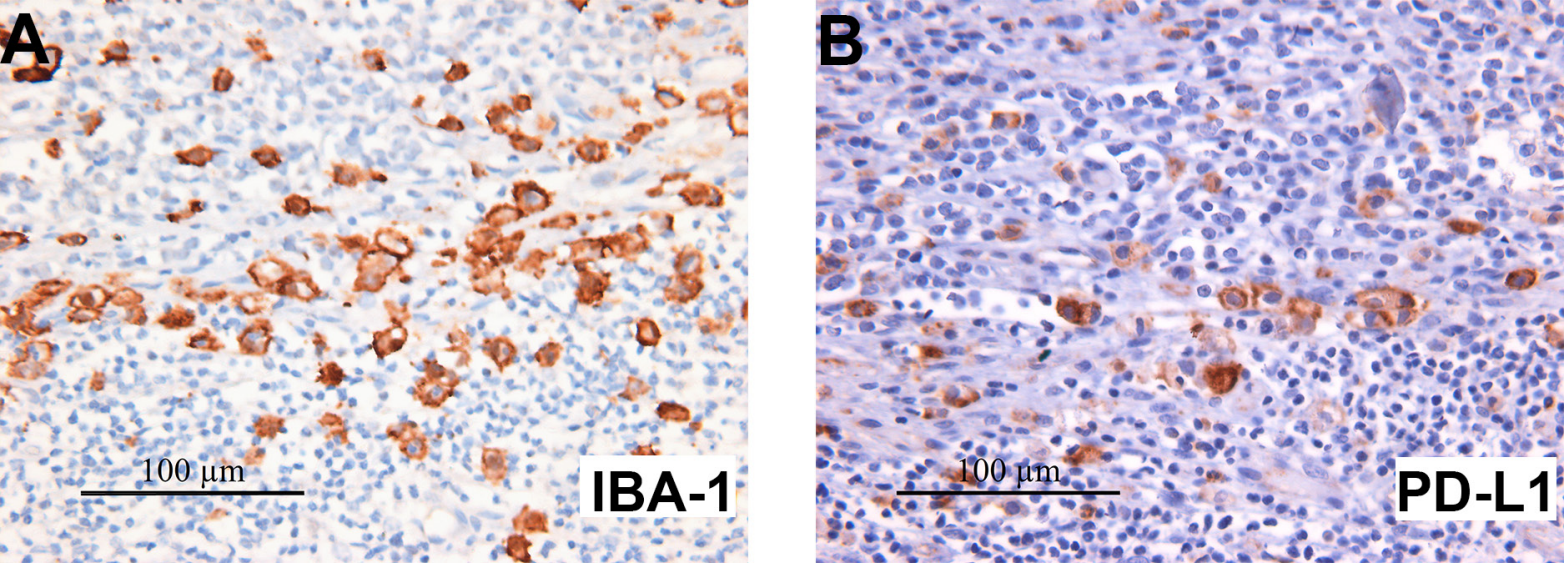
IBA-1 staining reveals heavy microglial infiltration in SHH MB with many microglia co-expressing PD-L1 Immunohistochemistry of one representative SHH MB, 18872, stained for IBA-1 (**A**) and PD-L1 (**B**). Many of the IBA-1 expressing microglial cells are also PD-L1+. All images 400× original magnification.

Using 2 cohorts of human MB tumors, we evaluated the degree of PD-L1 expression in relation to infiltrating immune cells. Initial screening for PD-L1 using an MB tumor tissue microarray from the Children’s Hospital of Philadelphia (CHOP) did not reveal any positive PD-L1 staining. However, when more extensive tissue samples were evaluated from cases on the array, there were areas of clear membranous PD-L1 expression in both the tumor cells and the infiltrating immune cells leading us to expand this inquiry to additional cases. Unfortunately, adequate tumor tissue to obtain fresh cut slides was only available for a limited number of cases. In addition to PD-L1 staining, tumor-infiltrating immune cells were identified by IHC as CD3+ tumor infiltrating lymphocytes (TIL) and CD68+ tumor infiltrating myeloid cells (TIM). In the Johns Hopkins Hospital (JHH) cohort, IBA-1 expression was also evaluated as a marker of microglia (Figure 1). Although the overall percentage of cells displaying PD-L1 was low across all subgroups, the tumor with the highest degree of tumoral PD-L1 expression, >2%, was in the SHH group. This case, #36, had an immune infiltrate of CD3+ cells present in the areas of highest PD-L1 expression, supporting a component of adaptive resistance via PD-L1 (Figure 2). Although the number of cases in this study was too small to determine a statistical trend, there were more cases with moderate TIL/TIM infiltrate in the SHH group as compared to Group 3/4 (Table 1). Increased density of IBA-1 expressing cells was also noted in the SHH tumors, some of which co-express PD-L1 (Figures 1 and 3, Table 1). Since dominant negative mutations in the *TP53* tumor suppressor gene are common in SHH MB and this mutation has been linked to increased PD-L1 expression in NSCLC, [17–19] we sought to determine whether mutant p53 was the cause of the increased PD-L1 expression in Case 36. The presence of mutant p53 protein expression was evaluated by IHC for all SHH cases. However only one of the SHH cases, #18861, expressed mutant p53 protein, and there was no PD-L1 expression or immune infiltrate noted in this case (Table 1). One WNT MB case was available for review, #18905, which demonstrated no tumoral PD-L1 expression and heavy infiltration of IBA-1+ cells (Table 1, Supplementary Figure 2).

**Table 1:** Degree of PD-L1 expression and immune infiltration by subgroup

Case	PD-L1 Tumor	TIL/TIM (CD3/CD68)	IBA-1 (microglia)	Subgroup	Cohort
**18905**	0%	1	3	WNT	JHH
**36**	>2%	2	NA	SHH	CHOP
**18831**	1%	2	3	SHH	JHH
**18840**	0%	0	2	SHH	JHH
18861^**^	0%	0	3	SHH	JHH
**18872**	0%	1	3	SHH	JHH
**18877**	1%	1	3	SHH	JHH
**18881**	0%	2	2	SHH	JHH
**20**	0–1%	1	NA	3/4	CHOP
**25**	1–2%	1	NA	3/4	CHOP
**30**	0–1%	1	NA	3/4	CHOP
**40**	0–1%	1	NA	3/4	CHOP
18851^*^	0%	1	2	3/4	JHH
18870^*^	0%	1	2	3/4	JHH
18882^*^	0%	0	2	3/4	JHH
61379^*^	0%	2	3	3/4	JHH

PD-L1 expression, degree of CD3+ and CD68+ tumor infiltrating immune cells, IBA-1 expression, and MB subgroup for the combined cohorts.

^*^indicates Group3 designation.

^**^indicates presence of mutant p53.

### Medulloblastoma human tumor cell lines express the ligands of PD-1 in a subgroup dependent manner

**Figure 4:**
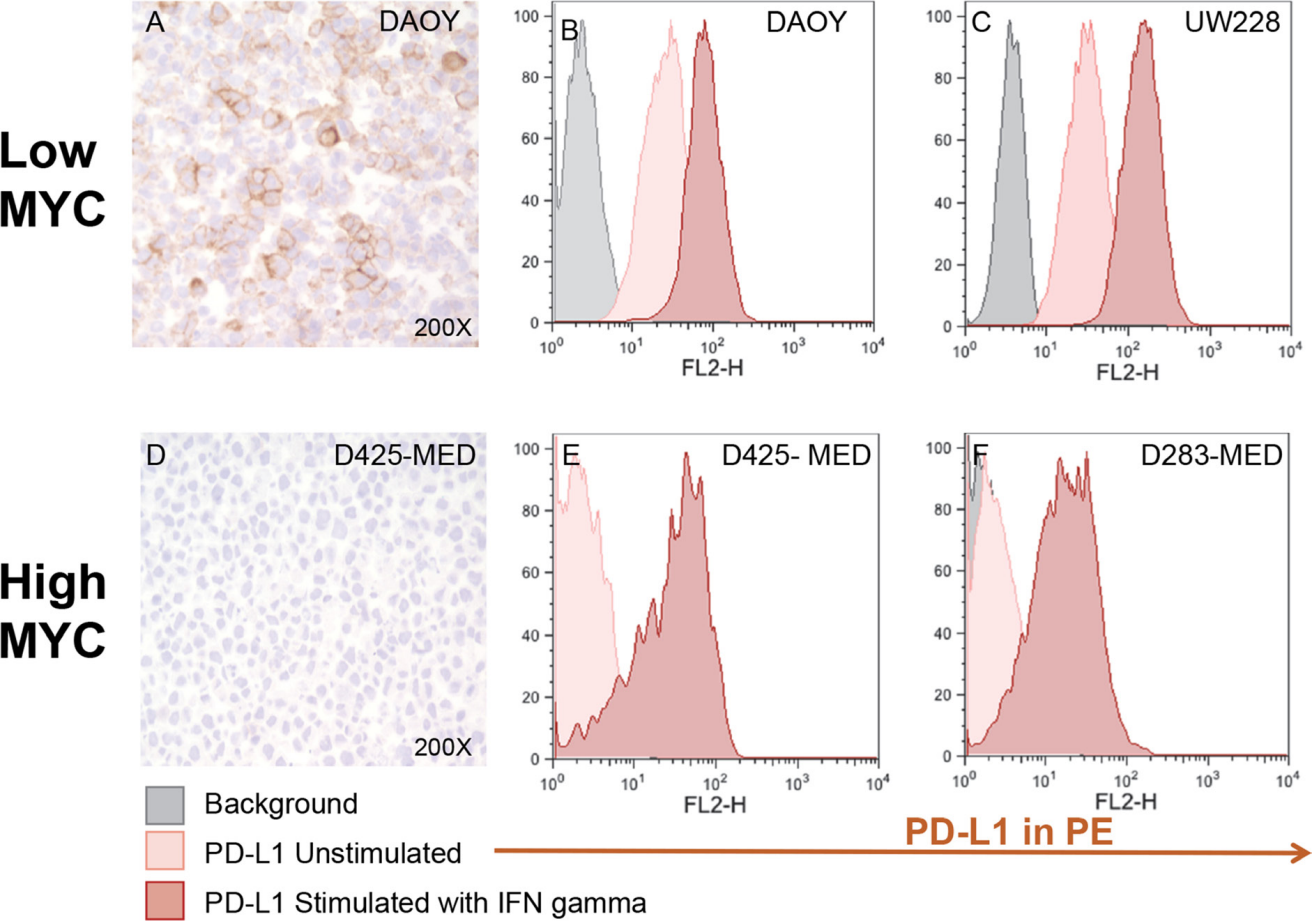
Medulloblastoma cell lines express PD-L1 in a subgroup dependent manner PD-L1 expression in low and high MYC MB cell lines. Paraffin embedded cell line pellets stained for PD-L1 in DAOY (**A**) and D425-MED (**D**). Histograms of PD-L1 expression by flow cytometry using phycoerythrin (PE) conjugated MIH1 clone of PD-L1 (EBioscience) with and without IFN-γ stimulation in DAOY (**B**), UW228 (**C**), D425-MED (**E**), and D283-MED (**F**).

To further explore the expression of PD-L1 in MB in relation to molecular subgroup, we sought to determine whether expression levels might be intrinsically related to molecular characteristics of the tumors *in vitro* - or if PD-L1 expression (or lack therof) was primarily a reaction to factors in the local tumor microenvironment. To do this we evaluated 4 extensively characterized human MB cell lines, DAOY, UW228, D283-MED, and D425-MED. D283-MED and D425-MED are known to harbor high levels of MYC most consistent with the aggressive Group 3 tumors although D283-MED has been classified as having molecular features of both Group 3 and Group 4 [20, 21]. DAOY and UW228 are not MYC amplified and are more similar to SHH tumors [22, 23]. Expression of PD-1’s ligands, PD-L1 and PD-L2, were evaluated at rest and after 48 hours of stimulation with the T_H_1 cytokine IFN-γ. Strikingly, a distinct pattern of expression was noted. The low MYC tumors, DAOY and UW228, expressed a significant amount of PD-L1 at rest and then further responded to IFN-γ stimulation, displaying both intrinsic and inducible expression patterns (Figure 4). Conversely, the MYC amplified tumors, D283-MED and D425-MED, only expressed PD-L1 in response to IFN-γ (Figure 4). This pattern was confirmed over the course of 5 individual experiments and analyzed as a function of median fluorescence intensity (MFI), total percent PD-L1 positive cells, and change in percent PD-L1 positive cells with and without IFN-γ (Table 2, Supplementary Figure 3). PD-L2, the second ligand of PD-1, was expressed in an identical pattern to PD-L1 in the low MYC cell lines but was not expressed at all by the MYC amplified tumors either at rest or with stimulation (Supplementary Figure 4). To determine whether DAOY and UW228 were intrinsically more immunogenic or if this was specific to the PD-1 pathway, we also evaluated these tumors for the expression of human MHC class II ligands; DR, DQ, and DB. MB has been previously described as having the ability to express MHC class II [24–26], and indeed all cell lines expressed it in response to stimulation except for D283-MED (Supplementary Figure 5). No cell lines displayed intrinsic expression of MHC class II, consistent with prior reports, suggesting that the observed pattern of PD-L1 and PD-L2 was unique to molecules of the PD-1 pathway.

**Table 2:** Degree of PD-L1 expression across cell lines

	DAOY			UW228			D425-MED			D283-MED		
	Baseline	IFN-γ		Baseline	IFN-γ		Baseline	IFN-γ		Baseline	IFN-γ	
**Mean MFI PD-L1 positive cells ± SD**	19.4 ± 4.473	56.76 ± 23.44		15.84 ± 4.525	55.8 ± 21.84		12.73 ± 5.563	23.58 ± 17.17		8.844 ± 2.321	11.02 ± 2.796	
***P* value Welch’s *t*-test**	0.0196			0.0136			0.2385			0.2182		
**Mean percentage PD-L1 positive cells ± SD**	72.24 ± 20.08	88.26 ± 9.903		84.4 ± 10.32	95.08 ± 4.868		3.604 ± 4.098	55.84 ± 4.604		2.956 ± 1.09	42.28 ± 12.57	
***P* value Welch’s *t*-test**	0.1621			0.0838			0.0001			0.0018		
**Mean percentage PD-L1 positive above baseline ± SD**	2.046 ± 0.605		40.62 ± 20.31	1.246 ± 0.6954		50.19 ± 30.58	1.732 ± 0.4427		48.18 ± 13.26	1.308 ± 0.8933		35.74 ± 16.41
***P* value Welch’s *t*-test**	0.0132			0.0232			0.0014			0.0016		

PD-L1 is quantified using flow cytometry with and without IFN-γ stimulation and measured as a function of MFI, total percentage of PD-L1 positive cells, and percentage of PD-L1 positive cells above baseline expression.

### Elevated MYC expression alone is insufficient to suppress constitutive expression of PD-L1

**Figure 5:**
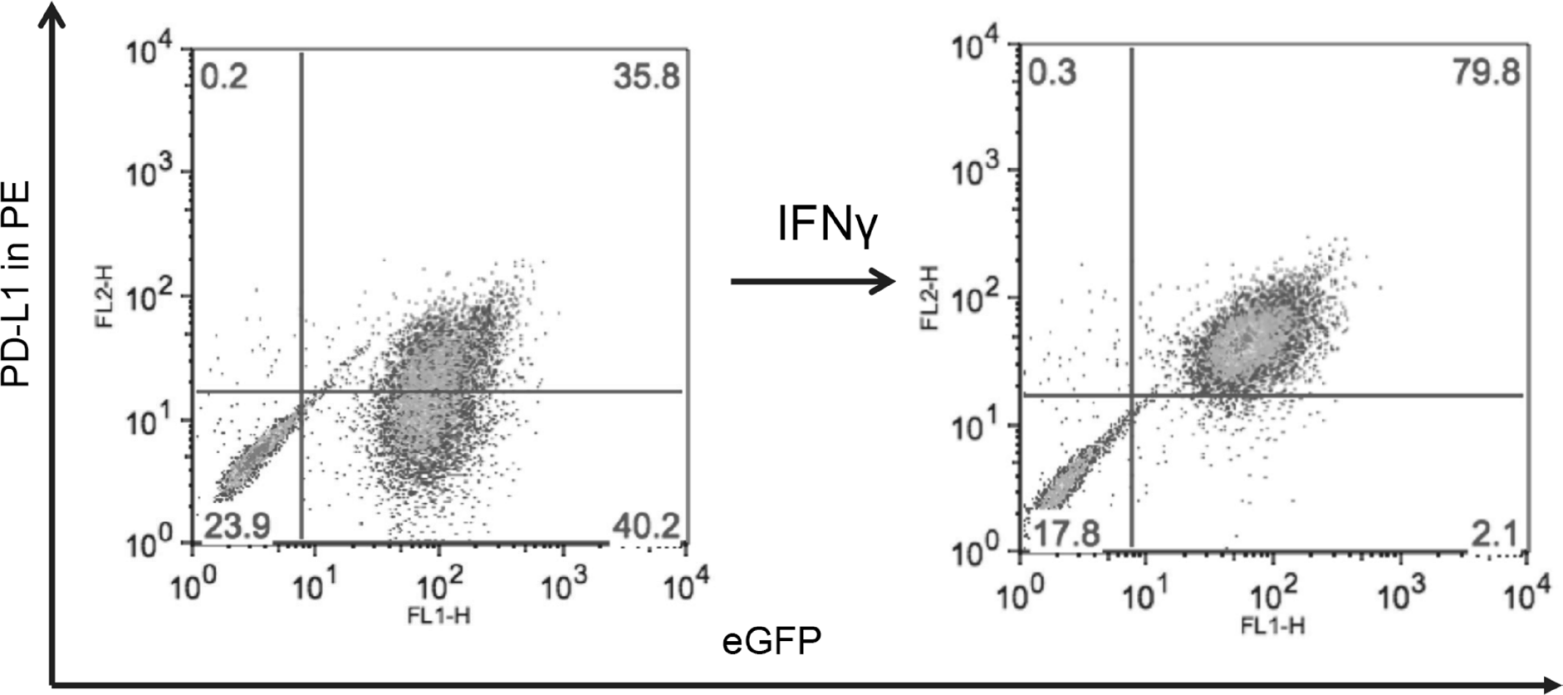
MYC overexpression in DAOY (YM21) does not alter PD-L1 expression Dot Plots of PD-L1 expression by flow cytometry using PE-conjugated MIH1 clone of PD-L1 (EBioscience) in YM21 construct made by overexpressing MYC in DAOY via stable lentiviral transfection.

Since high MYC expression in MB cell lines was negatively associated with the constitutive expression of PD-L1 and PD-L2 *in vitro*, we sought to evaluate whether MYC expression status could be a determinate of PD-L1 expression in MB. Therefore, we evaluated previously validated isolates of DAOY and UW228 stably transduced with MYC overexpression plasmids, YM21 and UWM13 [27], respectively, to see if this would suppress immune checkpoint ligand expression in these cell lines. However, MYC overexpression did not alter the pattern of PD-L1 expression, indicating that overexpression of MYC was not sufficient to alter the constitutive expression of PD-L1, and that this correlation may be regulated by another pathway (Figure 5).

### Radiation is not as potent an inducer of PD-L1 expression as IFN-γ in medulloblastoma cells

**Figure 6:**
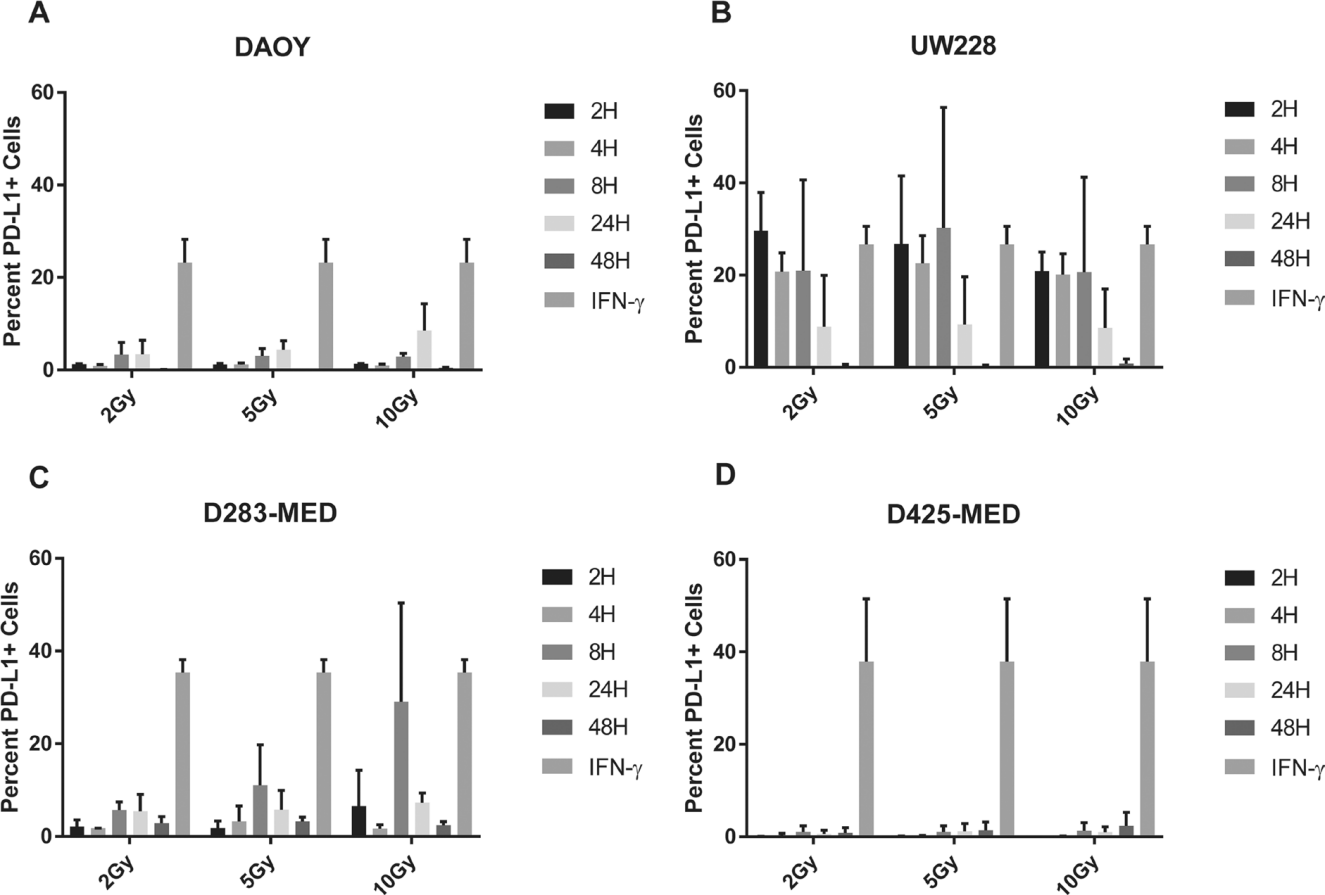
Radiation induces PD-L1 in most medulloblastoma cell lines Bar graph depicts percent PD-L1 positive cells above baseline as determined by flow cytometry using PE-conjugated MIH1 clone of PD-L1 (EBioscience) in DAOY (**A**), UW228 (**B**), D283-MED (**C**), and D425-MED (**D**) after irradiation with 2, 5, or 10 Gy at 2, 4, 8, 24, and 48 hours. IFN-γ values were obtained without radiation. Each bar graph depicts findings from 2 different experiments where all data points were repeated. Error bars represent standard deviation of the mean. Levels of PD-L1 that were comparable to those induced by IFN-γ included UW228 hours 2–24 at 2, 5 and 10 Gy and D283-MED at hour 8, 10 Gy. All other values were significantly less than those induced by IFN-γ as determined by 2-way ANOVA corrected for multiple comparisons. Full statistical analysis available in Supplementary Table 1-1–1-4.

Both clinical and pre-clinical studies indicate additive value to combining radiation therapy with PD-1 or PD-L1 blockade, and it has been postulated that radiation induces PD-L1 expression on tumors based on *in vivo* modeling data [28–30]. In addition, radiation is a regularly used treatment for patients with MB, so understanding the immune effects is critical. To determine whether the up-regulation of PD-L1 we noted in MB could be induced by radiation, we evaluated the PD-L1 response of MB tumor cell lines post radiation. Using a high energy X-ray radiator, cell lines in culture were exposed to 0, 2, 5, or 10 Gy. PD-L1 expression was assessed by flow cytometry 2, 4, 8, 24, and 48 hours later. 2 Gy was chosen as a starting dose since this approximates a typical daily fraction during standard of care radiation therapy for MB [31]. The percent change in PD-L1 expression above baseline for each cell line was analyzed at the various radiation doses and time points and compared concomitantly to PD-L1 expression induced by IFN-γ (Figure 6). Radiation was able to induce PD-L1 expression to some degree in all cell lines, but rarely to the extent seen with IFN-γ. PD-L1 response to radiation was variable across cell lines, but the patterns were strikingly similar regardless of radiation dose (Figure 6). UW228 was the cell line most responsive to radiation and demonstrated comparable induction of PD-L1 to IFN-γ at every time point except 48 hours. PD-L1 expression peaked at 8H post all dose levels of radiation in D283-MED but this was only statistically comparable to IFN-γ after 10Gy XRT. DAOY demonstrated highest PD-L1 induction 24H post XRT but this did not approach the level of induction seen with IFN-γ and remained statistically different. Interestingly, there was an overall down regulation of PD-L1 by 48 hours in all cell lines except D425-MED where there was minimal response to radiation at any time point (Figure 6, Supplementary Table 1-1–1-4).

## DISCUSSION

There continues to be great excitement surrounding the potential for immune checkpoint inhibitor therapy to treat an ever-expanding array of oncologic disorders. At the same time, there has been mounting frustration regarding the inability to predict clinical responses to these agents. In most studies, increased levels of tumoral PD-L1 expression have correlated with therapeutic anti-tumor responses to PD-1 blockade as was recently reviewed by Sunshine and Taube [32]. However, there have also been patients without PD-L1 biomarker expression who have benefited clinically from PD-1 pathway blockade [14, 33–35]. Additionally, most patients are assessed for PD-L1 expression prior to starting therapy, but the expression of PD-L1 during the entire course of treatment remains unclear, as does the relationship between changing PD-L1 expression and therapeutic responses. Therefore, there is a pressing need to better understand how intrinsic tumoral factors influence the dynamic expression of PD-L1 in different tumors as well as corresponding microenvironment features that might also be predictive of a clinical response. In this study, we found a low level of PD-L1 expression in MB human tumor samples, with most samples demonstrating PD-L1 on 1% of cells or fewer. *In vitro* cell line studies indicated higher levels of PD-L1 in SHH MB versus Group 3/4; however, we could not confirm a clear association between SHH MB and increased markers of inflammation in human samples. Several recent studies have also evaluated PD-L1 expression in MB with varied results. A large study by Majzner *et al.* evaluated 40 pediatric MB samples but did not find any expressing PD-L1 [36], while a study by Murata *et al.* reported “high” PD-L1 expression in 9/16 MB cases [37]. Another study from the Netherlands reported no PD-L1 expression for 26 MB cases [38]. The reason for the discrepancy in expression across different studies is likely attributable to differences in antibody sensitivity and staining conditions, as has been shown for a variety of adult diseases [12, 14, 33–35, 39]. Our study utilized a manual staining and scoring technique optimized for the detection of PD-L1 expression to increase sensitivity while still maintaining specificity by using a monoclonal antibody and comparing each case to a separate IgG isotype control. We also directly quantified any PD-L1 positivity rather than setting an arbitrary threshold for positivity. Our findings further emphasize the difficulty in quantifying biomarker expression in this pathway and the need to standardize assays and scoring cut-offs. In support of PD-L1 pathway activity in human MB, we demonstrated that MB cell lines robustly up-regulated PD-L1 when we simulated an anti-tumor immune response *in vitro* by exposing the cell lines to recombinant human IFN-γ. This is the classic response pattern demonstrated in melanoma and is characteristic of adaptive immune resistance whereby tumor cells respond to immunologic attack by expressing an inhibitory immune checkpoint molecule [13, 40]. These data suggest that in the setting of an anti-tumor immune response, MB, like melanoma, can utilize the ligands of PD-1 to evade detection and destruction by the immune system. Consistent with prior observations, we found an overall paucity of infiltrating immune cells in our MB cohorts suggesting that a robust anti-tumor immune response is not occurring in MB at the time of diagnostic surgical resection [38, 41]. However, in support of adaptive immune resistance, areas of the tumor with focally increased lymphocytic infiltrate did indeed express higher levels of tumoral PD-L1 (Figure 2). In further support of the notion that MB adaptively up-regulates PD-L1 as a specific response to immune mediated stimulation is the finding that radiation induced PD-L1 expression but not to the extent generated by IFN-γ. Radiation is known to induce the expression of tumoral PD-L1 in *in vivo* studies [28]. Our findings suggest that this induction could be further mediated by cytokine release from infiltrating immune cells in response to radiation-induced damage in addition to direct radiation effects on tumor cells. The finding that both IFN-γ and radiation induced PD-L1 expression *in vitro* and the paucity of PD-L1 expression *in vivo* in the absence of TIL further emphasizes the concept that immune adjuvants will likely be needed to fully realize the benefit of PD-1 blockade in “cold” tumors such as MB [10].

Previous work from members of our group found that radiation treatment drives tumor infiltration by T cells *in vivo* and that specific anti-tumor activation is augmented greatly by combining radiation with PD-1 blockade [30]. Radiation demonstrated synergy with PD-1 blockade in several other pre-clinical models [29, 42] and has been used clinically in combination with anti-PD-1 for patients with melanoma brain metastases, although the results of prospective randomized clinical trials are lacking [43, 44]. Since radiation is already a mainstay of upfront MB therapy and frequently used in the salvage setting, we feel this could be a logical therapeutic approach in this tumor and should be considered for future studies. Importantly, we found that PD-L1 induction, when it occurred, happened within the first 24 hours post radiation with PD-L1 being significantly down-regulated in most cell lines by 48 hours. This suggests that the timing of PD-1 blockade may be important for the patient undergoing radiation therapy, however this finding needs to be validated with *in vivo* studies before translating to the clinic. Although the greatest induction of PD-L1 occurred after exposure to 10 Gy radiation, similar trends were noted at 2 and 5 Gy. We could not fully simulate the typical 54 Gy given over 6 weeks used for newly diagnosed MB in an *in vitro* study; however, the changes noted at just 2 Gy could have implications for patients undergoing typical fractionated radiotherapy [31]. 10 Gy approximates a stereotactic radiation dose that might be used in the setting of focally relapsed MB and has been shown by our group to be an effective strategy in a mouse model of glioma [29].

Combining multiple immune checkpoint inhibitors is another strategy frequently employed to potentiate the anti-tumor immune response in patients with low levels of PD-L1 expression or who are resistant to PD-1 pathway blockade [45–49]. Pham *et al.*, tested the combination of anti-PD-1 with anti-CTLA-4 in murine models of both SHH and Group 3 MB. Interestingly, although the combination was active against both subgroups, the MYC amplified MB model had a more inflamed TME and a better response to immune checkpoint inhibition than did the SHH MB model [8]. These results suggest that the relatively low immunogenicity of MB might be overcome by using multiple modalities to stimulate the immune system. Although PD-L1 expression is linked to response to monotherapy with PD-1 blockade, this has been far less predictive of responses to PD-1 blockade combined with CTLA-4 blockade. In fact, many tumors with expression levels of 1% or less have demonstrated objective clinical responses to this combination [47, 49]. This disparity likely reflects the differing mechanism of action for these two immune inhibitory receptors, with CTLA-4 affecting the interaction between T cells and antigen presenting cells (APCs) allowing for widespread immune activation beyond the TME including at the level of the draining lymph node as opposed to PD-1’s more localized tumor effects [10]. The therapeutic combination of anti-PD-1 and anti-CTLA-4 is now available for relapsed MB patients for the first time in the newly opened therapeutic trial, NCT03130959, so additional clinical information pertaining to this hypothesis in MB will be forthcoming.

Expression of MHC II by MB is unusual as this molecule is usually a feature of dendritic cells and other APCs indicating that this tumor may be directly inhibiting anti-tumor immune responses by masquerading as an inhibitory APC [24, 50] MHC II expression may also indicate a role for the immune checkpoint molecule, lymphocyte activating gene-3 (LAG-3) in MB whose primary ligand is MHC II. The exact mechanism of LAG-3 signaling remains unknown but was recently reviewed by Andrews *et al.* [51]. LAG-3 is known to inhibit APC activation, and in melanoma, MHCII expression was correlated with apoptotic resistance, possibly via LAG-3 engagement suggesting that anti-LAG-3 could be an important therapeutic strategy in MHC II expressing tumors [52, 53]. Anti-LAG-3 is emerging as an important clinical antibody in combination with anti-PD-1 based on pre-clinical studies indicating therapeutic syngergy, [54, 55] and is being evaluated currently for adults with recurrent GBM (NCT02658981). As yet there have not been any clinical trials of anti-LAG-3 in pediatric patients.

In conclusion, for children with brain tumors, immune based therapies hold the promise of potentially supplanting the more toxic standard-of-care approaches such as radiation and chemotherapy. Implementation of immune checkpoint blockade in MB would most likely occur in the relapsed/refractory setting since current treatment regimens are associated with a 5 year overall survival of 85% or higher [1]. However, it is now well known that MB is a heterogeneous disease characterized by at least four molecular subgroups. As we continue to better understand the clinical implications of subgroup designation in MB, upfront therapy will likely evolve to include risk stratification for patients based on these molecular features. Therefore, it is particularly important that we attempt to understand the way in which the molecular subgroup of MB influences immune checkpoint molecules and infiltrating immune cells to create unique tumor immune microenvironments that may respond differently to immune based therapies. This will be especially important to consider when both interpreting and designing clinical trials that incorporate immune-based treatments such as checkpoint inhibitors for the treatment of MB.

## MATERIALS AND METHODS

### Cell culture

Human MB cell lines were grown in a monolayer, DAOY, UW228, YM21, UWM13, or in suspension, D283-MED and D425-MED, under standard culture conditions with 5% CO2 in a 37° C incubator. All cell lines were kindly provided by Dr. Charles G Eberhart, MD, PhD (Johns Hopkins University, Baltimore, MD, USA) with the exception of D283-MED which was purchased directly from American Type Culture Collection (ATCC) (Manassas, VA, USA). All tissue culture media was supplemented with 10% fetal bovine serum and 1% penicillin/streptomycin. DAOY, D283-MED, and D425-MED were grown in MEM Medium (Invitrogen, Grand Island, New York, USA) and additionally supplemented with 10% sodium pyruvate and non-essential amino acids (Invitrogen, Grand Island, New York, USA); UW228 was grown in DMEM F-12 Medium (Invitrogen, Grand Island, New York, USA). Cell lines have been previously described including YM21 and UWM13 [27, 56–59]. STR testing for DAOY, UW228, D425-MED, YM21, UWM13 was performed by the Johns Hopkins Genetic Resources Core Facility (Johns Hopkins University, Baltimore, MD, USA) in January 2013. Mycoplasma testing was performed DAOY, UW228, D425-MED, UWM13, and YM21 using the MycoProbe™ Mycoplasma Detection Kit (R&D Systems, Minneapolis, MN, USA). Tested isolates were expanded and frozen in multiple aliquots for subsequent experiments. D283-MED was purchased directly from ATCC in June 2013 and frozen in multiple aliquots at low passage. No additional testing was performed.

For stimulation experiments, cells were plated at a density of ∼5,000/cm^2^ into 100 cm^2^ tissue culture dishes or T25 flasks and allowed to rest for 24 hours. After 24 hours, cell cultures were treated with recombinant human interferon gamma (IFN-γ) (300-02, Pepro Tech, Rocky Hill, Connecticut, USA) at a concentration of 100 units/mL for 48 hours prior to harvest. All stimulation experiments were repeated at least 5 times. For radiation experiments cells were plated at a density of ∼5,000/cm^2^ into 100 cm^2^ tissue culture dishes or T25 flasks and allowed to rest for 24 hours. They were irradiated using a high energy X-ray radiator to either 0, 2, 5 or 10 Gy. They were returned to the incubator and harvested for at 2, 4, 8, 24, and 48 hours post radiation exposure for flow cytometry. All radiation experiments were repeated 2 times.

### Flow cytometry

Cells were stained with LIVE/DEAD^®^ Fixable Far Red Dead Cell Stain Kit, for 633 or 635 nm excitation (Fisher Scientific) at a concentration of 1:1000 at room temperature (RT) in PBS for 30 min. They were subsequently washed and then stained with either phycoerythrin (PE) labeled mouse anti-human PD-L1 (CD274, clone MIH 1, 12-5983-41, Ebiosciences, San Diego, CA, USA), PE-labeled mouse anti-human PD-L2 (CD273, clone MIH18 clone, 12-5888-41, Ebiosciences, San Diego, CA, USA), or fluorescein (FITC) labeled mouse anti-human HLA DR, DP, DQ (clone Tu39, 555558, Becton Dickinson, Franklin Lakes, New Jersey, USA). Antibodies were diluted 1:200 and staining was performed in FACS buffer or PBS for 15 minutes at RT. The same procedure was performed using isotype control antibodies, PE-labeled mouse IgG1 kappa or FITC-labeled mouse IgG2a kappa. Cells were analyzed using a BD FACS Calibur (Becton Dickinson, Franklin Lakes, New Jersey, USA) and FlowJo software (Tree Star, Ashland, Oregon, USA). Manual gating was used to determine PD-L1 positive thresholds. Due to the differential expression patterns in the 4 cell lines, changes were not well quantified by MFI or total percent PD-L1 positive cells across all 4 cell lines. Therefore, for quantitative analysis percent PD-L1 positive cells above baseline was used as a metric. Examples of gating strategies for each cell line are provided in Supplementary Figure 3. Flow cytometric analyses were repeated 2–5 times.

### Human medulloblastoma samples

An MB/supratentorial CNS embryonal tissue microarray with 44 tumors as well as 5 paraffin embedded primary MB specimens were obtained from a brain tumor database maintained by the Department of Pathology at the Children’s Hospital of Philadelphia (CHOP) under IRB approved protocol, 13-010191. 11 paraffin embedded primary MB specimens were obtained from a brain tumor database maintained by the Department of Pathology at the Johns Hopkins Hospital (JHH) under IRB approved protocol NA_00015113.

### Immunohistochemistry

#### Human tumors

Using 2 cohorts of human MB tumors, we evaluated the degree of PD-L1 expression in relation to infiltrating immune cells by subgroup. Immunohistochemistry (IHC) for PD-L1 (5H1 clone, courtesy of Lieping Chen, MD, PhD) was performed as previously described [13] for the 5 individual cases and a 44 MB sample tissue microarray provided by CHOP. Briefly, slides were de-paraffinized and rehydrated in xylenes and a graded series of alcohols. Antigen retrieval was performed in pH9.0 TE buffer in a de-cloaking chamber (Biocare Medical, Pacheco, CA, USA). Followed by peroxidase, protein, avidin and biotin block. The primary antibody at 1.8 ug/uL concentration was incubated overnight at 4° C. Biotin labeled anti-mouse secondary antibody was used at 1 µg/µL concentration followed by amplification (K1500 Dako Agilent, Santa Clara, CA, USA). Signal was visualized by DAB. IHC for PD-L1 (SP142 clone, Spring Bioscience, Pleasanton, CA, USA) was performed on a second cohort of patient tumors from JHH [60]. Briefly, slides were de-paraffinized and rehydrated in xylenes and a graded series of alcohols. Antigen retrieval was performed in pH6.0 CB buffer in a de-cloaking chamber (Biocare Medical, Pacheco, CA, USA). Followed by peroxidase, protein, avidin and biotin block. The primary antibody at 0.096 ug/mL concentration was incubated overnight at 4° C. Biotin labeled anti-rabbit secondary antibody was used at 1 µg/µL concentration. Signal was developed using the Vectastain Elite ABC kit (PK-6100 Vector Laboratories, Burlingame, CA, USA) followed by amplification with the Perkin Elmer tyramide signal amplification plus biotin kit (dilution 1:50). Signal was visualized by DAB. The anti-PD-L1 clones SP142 and 5H1 have been shown to have comparative analytic performance for the quantification of PD-L1 expression when used in the protocols described above [61]. Positive and negative controls for this assay were accomplished using human tonsil: paracortical histiocytes act as a positive control for PD-L1 while lymphocytes act as a negative control [62]. Each MB slide stained for PD-L1 was paired with a slide stained with the corresponding isotype antibody to control for non-specific antibody binding (Supplementary Figure 1).

Tumors from the CHOP cohort were assigned to one of 3 molecular subgroups according to the St Jude protocol for immunohistochemical subgrouping published by Ellison *et al.* to identify WNT, SHH and Group 3/4 [5]. Staining for YAP1, GAB1, and nuclear beta catenin was performed in the Johns Hopkins Clinical Pathology Core Lab using automated CLIA certified staining protocols for these antibodies. Tumors in the JHH cohort had been previously assigned to a molecular subgroup by the Institute of Pathology at the University of Heidelberg using a different immunohistochemical panel that has been previously described to identify 4 distinct subgroups, WNT, SHH, Group 3, and Group 4 [2].

Tumors from the CHOP cohort were stained for mutant p53 protein with the D0-7 mouse anti-human monoclonal p53 antibody (M7001 Dako Agilent, Santa Clara, CA, USA) [63]. Primary antibody clone D0-7 (M7001 Dako Agilent, Santa Clara, CA, USA) was used with the pretreatment protocol E2-20 in a dilution of 1:100 for one hour at room temperature using a Refine Detection staining kit on a Bond Max autostainer (Leica, Buffalo Grove, Illinois, USA). Tumors from the JHH cohort were stained for mutant p53 protein with the BP-53–12 mouse anti-human monoclonal p53 antibody BP-53 (P5813 Sigma Aldrich, Saint Louis, MO, USA) [64] using a standardized Ventana protocol. Positive and negative controls for this staining were accomplished using human tumors harboring known somatic dominant negative *TP53* mutations from hosts with wildtype germline *TP53*. Thus tumor cells acted as positive controls and stromal tissue as negative controls.

IHC for CD3, CD68, and IBA-1 on all samples was performed in the Johns Hopkins Clinical Pathology Core Laboratory using automated CLIA certified staining protocols for these antibodies.

#### Human tumor cell lines

The 5H1 clone was also used to identify PD-L1 staining on tumor cell line pellets. Cell line pellets were generated by the Johns Hopkins Oncology Tissue Services Core Facility by formalin fixing cell line pellets and then embedding in paraffin. After that staining was performed the same as above.

#### Scoring

PD-L1 and the degree of CD3+, CD68+, and IBA-1+ immune cell infiltrates were scored as previously described [13] by a board-certified pathologist, JMT. Briefly, tumor cells exhibiting membranous PD-L1 expression were reported as a percentage, and a semi-quantitative intensity grade was assigned to the infiltrating immune cells of 0 (none), 1 (mild), 2 (moderate), and 3 (severe) [13]. CHOP samples were assigned a subgroup designation to one of three subgroups, WNT, SHH, or Group 3/4 by another board certified pathologist, CGE. Subgroup designation by IHC has been previously described [5]. Subgroup assignment for the JHH cohort was performed previously at the University of Heidelberg using their previously described IHC method and these results have been previously published [2]. Scoring of mutant p53 staining on the CHOP cohort was performed by board certified pathologist, MS, and mutant p53 scoring of the JHH cohort was performed by board certified pathologist, CGE.

#### Imaging

IHC imaging was performed using a ProgRes^®^ C14plus microscope camera with a CCD color sensor with up to 12.5 megapixels resolution. All images are 8 bit. Post processing performed using Adobe Photoshop CS6 and included white balancing and resizing only.

#### Statistics

All statistical calculations were performed using Prism (GraphPad Software, SanDiego, CA, USA). The degree of immune cell infiltrates and PD-L1 expression in human tumors was compared using an unpaired, non-parametric, Mann-Whitney test to compare the median values in SHH versus Group 3/4. Where values were expressed as a range, the mid number in the range was assigned. The change in PD-L1 expression in cell lines after IFN-γ stimulation was quantified three ways: median fluorescence intensity (MFI) of PE conjugated PD-L1 in the FL-2H channel, total percentage of PD-L1 positive cells, and percentage of PD-L1 cells above baseline expression (Table 2). Paired Welch’s *t*-test was used to compare the values before and after IFN-γ for each cell line across 5 separate stimulation experiments. Two-way ANOVA evaluating radiation dose response as a function of time was used with Dunnet’s square test for multiple comparisons was used to compare the percentage of PD-L1 positive cells above baseline (no radiation, no stimulation) at each time point against the expression induced by IFN-γ. This analysis was performed separately for each cell line. Details of the statistical analysis are available in Supplementary Table 1-1–1-4.

## SUPPLEMENTARY MATERIALS FIGURES AND TABLES





## Abbreviations

ANOVA: analysis of variance
APC: antigen presenting cell
ATCC: American Type Culture Collection
CHOP: Children’s Hospital of Philadelphia
CLIA: Clinical Laboratory Improvement Amendments
CNS: central nervous system
CTLA-4: Cytotoxic T-lymphocyte-associated antigen 4
DAB-3,3′: diaminobenzidine
DMEM: Dulbecco’s Modified Eagle’s medium
FITC: fluorescein isothiocyanate
FACS: Fluorescence-activated cell sorting
GAB1: GRB2 Associated Binding Protein 1
Gy: gray
HLA: human leukocyte antigen
IBA-1: Ionized calcium binding adaptor molecule 1
IHC: immunohistochemistry
IFN: γ-interferon-gamma
IHC: immunohistochemistry
IRB: institutional review board
JHH: Johns Hopkins Hospital
LAG-3: lymphocyte activation gene-3
MB: medulloblastoma
MEM: Minimum Essential Media
MHC II: major histocompatibility complex 2
NSCLC: non-small cell lung cancer
PBS: phosphate buffered saline
PD: 1: programmed death one
PD-L1: programmed death ligand one
PD-L2: programmed death ligand 2
PE: phycoerthrin
RT: room temperature
SHH: sonic hedgehog
STR: short tandem repeats
TE: Tris EDTA
TIL: tumor infiltrating lymphocytes
TIM: tumor infiltrating macrophages
TME: tumor microenvironment
UTR: untranslated region
XRT: radiation
YAP1: yes- associated protein 1

## References

[1] Crawford JR, MacDonald TJ, Packer RJ (2007). Medulloblastoma in childhood: new biological advances. Lancet Neurol.

[2] Northcott PA, Korshunov A, Witt H, Hielscher T, Eberhart CG, Mack S, Bouffet E, Clifford SC, Hawkins CE, French P, Rutka JT, Pfister S, Taylor MD (2011). Medulloblastoma comprises four distinct molecular variants. J Clin Oncol.

[3] Taylor MD, Northcott PA, Korshunov A, Remke M, Cho YJ, Clifford SC, Eberhart CG, Parsons DW, Rutkowski S, Gajjar A, Ellison DW, Lichter P, Gilbertson RJ (2012). Molecular subgroups of medulloblastoma: the current consensus. Acta Neuropathol.

[4] Louis DN, Perry A, Reifenberger G, von Deimling A, Figarella-Branger D, Cavenee WK, Ohgaki H, Wiestler OD, Kleihues P, Ellison DW (2016). The 2016 World Health Organization Classification of Tumors of the Central Nervous System: a summary. Acta Neuropathol.

[5] Ellison DW, Dalton J, Kocak M, Nicholson SL, Fraga C, Neale G, Kenney AM, Brat DJ, Perry A, Yong WH, Taylor RE, Bailey S, Clifford SC, Gilbertson RJ (2011). Medulloblastoma: clinicopathological correlates of SHH, WNT, and non-SHH/WNT molecular subgroups. Acta Neuropathol.

[6] Zhukova N, Ramaswamy V, Remke M, Pfaff E, Shih DJ, Martin DC, Castelo-Branco P, Baskin B, Ray PN, Bouffet E, von Bueren AO, Jones DT, Northcott PA (2013). Subgroup-specific prognostic implications of TP53 mutation in medulloblastoma. J Clin Oncol.

[7] Margol AS, Robison NJ, Gnanachandran J, Hung LT, Kennedy RJ, Vali M, Dhall G, Finlay JL, Erdreich-Epstein A, Krieger MD, Drissi R, Fouladi M, Gilles FH (2015). Tumor-associated macrophages in SHH subgroup of medulloblastomas. Clin Cancer Res.

[8] Pham CD, Flores C, Yang C, Pinheiro EM, Yearley JH, Sayour EJ, Pei Y, Moore C, McLendon RE, Huang J, Sampson JH, Wechsler-Reya R, Mitchell DA (2016). Differential Immune Microenvironments and Response to Immune Checkpoint Blockade among Molecular Subtypes of Murine Medulloblastoma. Clin Cancer Res.

[9] Topalian SL, Taube JM, Anders RA, Pardoll DM (2016). Mechanism-driven biomarkers to guide immune checkpoint blockade in cancer therapy. Nat Rev Cancer.

[10] Sharma P, Allison JP (2015). The future of immune checkpoint therapy. Science.

[11] Chen L, Han X (2015). Anti-PD-1/PD-L1 therapy of human cancer: past, present, and future. J Clin Invest.

[12] Taube JM, Klein A, Brahmer JR, Xu H, Pan X, Kim JH, Chen L, Pardoll DM, Topalian SL, Anders RA (2014). Association of PD-1, PD-1 ligands, and other features of the tumor immune microenvironment with response to anti-PD-1 therapy. Clin Cancer Res.

[13] Taube JM, Anders RA, Young GD, Xu H, Sharma R, McMiller TL, Chen S, Klein AP, Pardoll DM, Topalian SL, Chen L (2012). Colocalization of inflammatory response with B7-h1 expression in human melanocytic lesions supports an adaptive resistance mechanism of immune escape. Sci Transl Med.

[14] Brahmer JR, Drake CG, Wollner I, Powderly JD, Picus J, Sharfman WH, Stankevich E, Pons A, Salay TM, McMiller TL, Gilson MM, Wang C, Selby M (2010). Phase I study of single-agent anti-programmed death-1 (MDX-1106) in refractory solid tumors: safety, clinical activity, pharmacodynamics, and immunologic correlates. J Clin Oncol.

[15] Herbst RS, Soria JC, Kowanetz M, Fine GD, Hamid O, Gordon MS, Sosman JA, McDermott DF, Powderly JD, Gettinger SN, Kohrt HE, Horn L, Lawrence DP (2014). Predictive correlates of response to the anti-PD-L1 antibody MPDL3280A in cancer patients. Nature.

[16] Ayers M, Lunceford J, Nebozhyn M, Murphy E, Loboda A, Kaufman DR, Albright A, Cheng JD, Kang SP, Shankaran V, Piha-Paul SA, Yearley J, Seiwert TY (2017). IFN-γ-related mRNA profile predicts clinical response to PD-1 blockade. J Clin Invest.

[17] Cortez MA, Ivan C, Valdecanas D, Wang X, Peltier HJ, Ye Y, Araujo L, Carbone DP, Shilo K, Giri DK, Kelnar K, Martin D, Komaki R (2015). PDL1 Regulation by p53 via miR-34. J Natl Cancer Inst.

[18] Xu C, Hua H, Chen T, Zhang W, Song G, Zhang Z (2017). PD-L1 is correlated with p53 expression in patients with lung adenocarcinoma. Int J Clin Exp Pathol.

[19] Muñoz-Fontela C, Mandinova A, Aaronson SA, Lee SW (2016). Emerging roles of p53 and other tumour-suppressor genes in immune regulation. Nat Rev Immunol.

[20] Bigner SH, Friedman HS, Vogelstein B, Oakes WJ, Bigner DD (1990). Amplification of the c-myc gene in human medulloblastoma cell lines and xenografts. Cancer Res.

[21] Snuderl M, Batista A, Kirkpatrick ND, Ruiz de Almodovar C, Riedemann L, Walsh EC, Anolik R, Huang Y, Martin JD, Kamoun W, Knevels E, Schmidt T, Farrar CT (2013). Targeting placental growth factor/neuropilin 1 pathway inhibits growth and spread of medulloblastoma. Cell.

[22] Triscott J, Lee C, Foster C, Manoranjan B, Pambid MR, Berns R, Fotovati A, Venugopal C, O’Halloran K, Narendran A, Hawkins C, Ramaswamy V, Bouffet E (2013). Personalizing the treatment of pediatric medulloblastoma: polo-like kinase 1 as a molecular target in high-risk children. Cancer Res.

[23] Ivanov DP, Coyle B, Walker DA, Grabowska AM (2016). In vitro models of medulloblastoma: choosing the right tool for the job. J Biotechnol.

[24] Stastny MJ, Brown CE, Ruel C, Jensen MC (2007). Medulloblastomas expressing IL13Ralpha2 are targets for IL13-zetakine+ cytolytic T cells. J Pediatr Hematol Oncol.

[25] Tamura K, Shimizu K, Yamada M, Okamoto Y, Matsui Y, Park KC, Mabuchi E, Moriuchi S, Mogami H (1989). Expression of major histocompatibility complex on human medulloblastoma cells with neuronal differentiation. Cancer Res.

[26] Etzell JE, Keet C, McDonald W, Banerjee A (2006). Medulloblastoma simulating acute myeloid leukemia: case report with a review of “myeloid antigen” expression in nonhematopoietic tissues and tumors. J Pediatr Hematol Oncol.

[27] Stearns D, Chaudhry A, Abel TW, Burger PC, Dang CV, Eberhart CG (2006). c-myc overexpression causes anaplasia in medulloblastoma. Cancer Res.

[28] Deng L, Liang H, Burnette B, Beckett M, Darga T, Weichselbaum RR, Fu YX (2014). Irradiation and anti-PD-L1 treatment synergistically promote antitumor immunity in mice. J Clin Invest.

[29] Zeng J, See AP, Phallen J, Jackson CM, Belcaid Z, Ruzevick J, Durham N, Meyer C, Harris TJ, Albesiano E (2013). Anti-PD-1 blockade and stereotactic radiation produce long-term survival in mice with intracranial gliomas. Int J Radiat Oncol Biol Phys.

[30] Sharabi AB, Nirschl CJ, Kochel CM, Nirschl TR, Francica BJ, Velarde E, Deweese TL, Drake CG (2015). Stereotactic Radiation Therapy Augments Antigen-Specific PD-1-Mediated Antitumor Immune Responses via Cross-Presentation of Tumor Antigen. Cancer Immunol Res.

[31] Slampa P, Zitterbart K, Dusek L, Ruzickova J, Magnova O, Coupek P, Hübnerova P, Ondrova B, Syptakova B (2006). Craniospinal irradiation of medulloblastoma in the supine position. Rep Pract Oncol Radiother.

[32] Sunshine J, Taube JM (2015). PD-1/PD-L1 inhibitors. Curr Opin Pharmacol.

[33] Hodi FS, Sznol M, Kluger HM, McDermott DF, Carvajal RD, Lawrence DP, Topalian SL, Atkins MB, Powderly JD, Sharfman WH, Puzanov I, Smith DC, Leming PD (2014). Long-term survival of ipilimumab-naive patients (pts) with advanced melanoma (MEL) treated with nivolumab (anti-PD-1, BMS-936558, ONO-4538) in a phase I trial. J Clin Oncol.

[34] Larkin J, Chiarion-Sileni V, Gonzalez R, Grob JJ, Cowey CL, Lao CD, Schadendorf D, Dummer R, Smylie M, Rutkowski P, Ferrucci PF, Hill A, Wagstaff J (2015). Combined nivolumab and ipilimumab or monotherapy in untreated melanoma. N Engl J Med.

[35] Reck M, Rodríguez-Abreu D, Robinson AG, Hui R, Csőszi T, Fülöp A, Gottfried M, Peled N, Tafreshi A, Cuffe S, O’Brien M, Rao S, Hotta K, KEYNOTE-024 Investigators (2016). Pembrolizumab versus chemotherapy for PD-L1–positive non–small-cell lung cancer. N Engl J Med.

[36] Majzner RG, Simon JS, Grosso JF, Martinez D, Pawel BR, Santi M, Merchant MS, Geoerger B, Hezam I, Marty V, Vielh P, Daugaard M, Sorensen PH (2017). Assessment of programmed death-ligand 1 expression and tumor-associated immune cells in pediatric cancer tissues. Cancer.

[37] Murata D, Mineharu Y, Arakawa Y, Liu B, Tanji M, Yamaguchi M, Fujimoto K, Fukui N, Terada Y, Yokogawa R, Yamaguchi M, Minamiguchi S, Miyamoto S (2018). High programmed cell death 1 ligand-1 expression: association with CD8+ T-cell infiltration and poor prognosis in human medulloblastoma. J Neurosurg.

[38] Vermeulen JF, Van Hecke W, Adriaansen EJ, Jansen MK, Bouma RG, Hidalgo JV, Fisch P, Broekhuizen R, Spliet WG, Kool M, Bovenschen N (2017). Prognostic relevance of tumor-infiltrating lymphocytes and immune checkpoints in pediatric medulloblastoma. Oncoimmunology.

[39] Patel SP, Kurzrock R (2015). PD-L1 Expression as a Predictive Biomarker in Cancer Immunotherapy. Mol Cancer Ther.

[40] Haile ST, Bosch JJ, Agu NI, Zeender AM, Somasundaram P, Srivastava MK, Britting S, Wolf JB, Ksander BR, Ostrand-Rosenberg S (2011). Tumor cell programmed death ligand 1-mediated T cell suppression is overcome by coexpression of CD80. J Immunol.

[41] Wood GW, Morantz RA (1979). Immunohistologic evaluation of the lymphoreticular infiltrate of human central nervous system tumors. J Natl Cancer Inst.

[42] Twyman-Saint Victor C, Rech AJ, Maity A, Rengan R, Pauken KE, Stelekati E, Benci JL, Xu B, Dada H, Odorizzi PM, Herati RS, Mansfield KD, Patsch D (2015). Radiation and dual checkpoint blockade activate non-redundant immune mechanisms in cancer. Nature.

[43] Ahmed KA, Stallworth DG, Kim Y, Johnstone PA, Harrison LB, Caudell JJ, Yu HH, Etame AB, Weber JS, Gibney GT (2016). Clinical outcomes of melanoma brain metastases treated with stereotactic radiation and anti-PD-1 therapy. Ann Oncol.

[44] Haymaker CL, Kim D, Uemura M, Vence LM, Phillip A, McQuail N, Brown PD, Fernandez I, Hudgens CW, Creasy C, Hwu WJ, Sharma P, Tetzlaff MT (2017). Metastatic Melanoma Patient Had a Complete Response with Clonal Expansion after Whole Brain Radiation and PD-1 Blockade. Cancer Immunol Res.

[45] Koyama S, Akbay EA, Li YY, Herter-Sprie GS, Buczkowski KA, Richards WG, Gandhi L, Redig AJ, Rodig SJ, Asahina H, Jones RE, Kulkarni MM, Kuraguchi M (2016). Adaptive resistance to therapeutic PD-1 blockade is associated with upregulation of alternative immune checkpoints. Nat Commun.

[46] Bu X, Mahoney KM, Freeman GJ (2016). Learning from PD-1 resistance: new combination strategies. Trends Mol Med.

[47] Escudier B, Tannir N, McDermott D, Frontera O, Melichar B, Plimack E, Barthelemy P, George S, Neiman V, Porta C, Choueiri TK, Powles T, Donskov F (2017). LBA5-CheckMate 214: Efficacy and safety of nivolumab plus ipilimumab (N plus I) v sunitinib (S) for treatment-naive advanced or metastatic renal cell carcinoma (mRCC), including IMDC risk and PD-L1 expression subgroups. Annals of Oncology.

[48] Harris SJ, Brown J, Lopez J, Yap TA (2016). Immuno-oncology combinations: raising the tail of the survival curve. Cancer Biol Med.

[49] Wolchok JD, Chiarion-Sileni V, Gonzalez R, Rutkowski P, Grob JJ, Cowey CL, Lao CD, Wagstaff J, Schadendorf D, Ferrucci PF, Smylie M, Dummer R, Hill A (2017). Overall survival with combined nivolumab and ipilimumab in advanced melanoma. N Engl J Med.

[50] Soos JM, Krieger JI, Stüve O, King CL, Patarroyo JC, Aldape K, Wosik K, Slavin AJ, Nelson PA, Antel JP, Zamvil SS (2001). Malignant glioma cells use MHC class II transactivator (CIITA) promoters III and IV to direct IFN-γ-inducible CIITA expression and can function as nonprofessional antigen presenting cells in endocytic processing and CD4(+) T-cell activation. Glia.

[51] Andrews LP, Marciscano AE, Drake CG, Vignali DA (2017). LAG3 (CD223) as a cancer immunotherapy target. Immunol Rev.

[52] Hemon P, Jean-Louis F, Ramgolam K, Brignone C, Viguier M, Bachelez H, Triebel F, Charron D, Aoudjit F, Al-Daccak R, Michel L (2011). MHC class II engagement by its ligand LAG-3 (CD223) contributes to melanoma resistance to apoptosis. J Immunol.

[53] Liang B, Workman C, Lee J, Chew C, Dale BM, Colonna L, Flores M, Li N, Schweighoffer E, Greenberg S, Tybulewicz V, Vignali D, Clynes R (2008). Regulatory T cells inhibit dendritic cells by lymphocyte activation gene-3 engagement of MHC class II. J Immunol.

[54] Grosso JF, Goldberg MV, Getnet D, Bruno TC, Yen HR, Pyle KJ, Hipkiss E, Vignali DA, Pardoll DM, Drake CG (2009). Functionally distinct LAG-3 and PD-1 subsets on activated and chronically stimulated CD8 T cells. J Immunol.

[55] Woo SR, Turnis ME, Goldberg MV, Bankoti J, Selby M, Nirschl CJ, Bettini ML, Gravano DM, Vogel P, Liu CL, Tangsombatvisit S, Grosso JF, Netto G (2012). Immune inhibitory molecules LAG-3 and PD-1 synergistically regulate T-cell function to promote tumoral immune escape. Cancer Res.

[56] He XM, Wikstrand CJ, Friedman HS, Bigner SH, Pleasure S, Trojanowski JQ, Bigner DD (1991). Differentiation characteristics of newly established medulloblastoma cell lines (D384 Med, D425 Med, and D458 Med) and their transplantable xenografts. Lab Invest.

[57] Keles GE, Berger MS, Srinivasan J, Kolstoe DD, Bobola MS, Silber JR (1995). Establishment and characterization of four human medulloblastoma-derived cell lines. Oncol Res.

[58] Jacobsen PF, Jenkyn DJ, Papadimitriou JM (1985). Establishment of a human medulloblastoma cell line and its heterotransplantation into nude mice. J Neuropathol Exp Neurol.

[59] Friedman HS, Burger PC, Bigner SH, Trojanowski JQ, Wikstrand CJ, Halperin EC, Bigner DD (1985). Establishment and characterization of the human medulloblastoma cell line and transplantable xenograft D283 Med. J Neuropathol Exp Neurol.

[60] Yanik EL, Kaunitz GJ, Cottrell TR, Succaria F, McMiller TL, Ascierto ML, Esandrio J, Xu H, Ogurtsova A, Cornish T, Lipson EJ, Topalian SL, Engels EA, Taube JM (2017). Association of HIV Status With Local Immune Response to Anal Squamous Cell Carcinoma: implications for Immunotherapy. JAMA Oncol.

[61] Sunshine JC, Nguyen PL, Kaunitz GJ, Cottrell TR, Berry S, Esandrio J, Xu H, Ogurtsova A, Bleich KB, Cornish TC, Lipson EJ, Anders RA, Taube JM (2017). PD-L1 Expression in Melanoma: A Quantitative Immunohistochemical Antibody Comparison. Clin Cancer Res.

[62] Dong H, Chen L (2003). B7-H1 pathway and its role in the evasion of tumor immunity. J Mol Med (Berl).

[63] Yaziji H, Massarani-Wafai R, Gujrati M, Kuhns JG, Martin AW, Parker JC (1996). Role of p53 immunohistochemistry in differentiating reactive gliosis from malignant astrocytic lesions. Am J Surg Pathol.

[64] Hanaford AR, Archer TC, Price A, Kahlert UD, Maciaczyk J, Nikkhah G, Kim JW, Ehrenberger T, Clemons PA, Dančík V, Seashore-Ludlow B, Viswanathan V, Stewart ML (2016). DiSCoVERing Innovative Therapies for Rare Tumors: Combining Genetically Accurate Disease Models with In Silico Analysis to Identify Novel Therapeutic Targets. Clin Cancer Res.

