# Supplementary material for: PD-L1 expression in medulloblastoma: an evaluation by subgroup
Source: Oncotarget. 2018 Apr 10;9(27):19177–91. doi: 10.18632/oncotarget.24951 (PMC5922386; doi:10.18632/oncotarget.24951)
Supplement: Supplementary file 1 [file oncotarget-09-19177-s001.pdf]

PD-L1 expression in medulloblastoma: an evaluation by subgroup

SUPPLEMENTARY MATERIALS

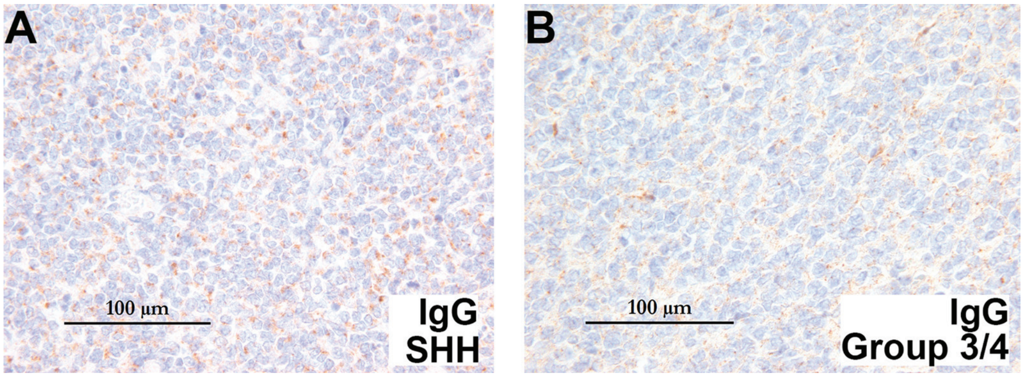

Supplementary Figure 1: Isotype antibody. mIgG1 Isotype Antibody to PD-L1 demonstrates minimal background staining in representative cases 36 (**A**) and 25 (**B**). Companion to Figure 1. Images 400× Magnification.

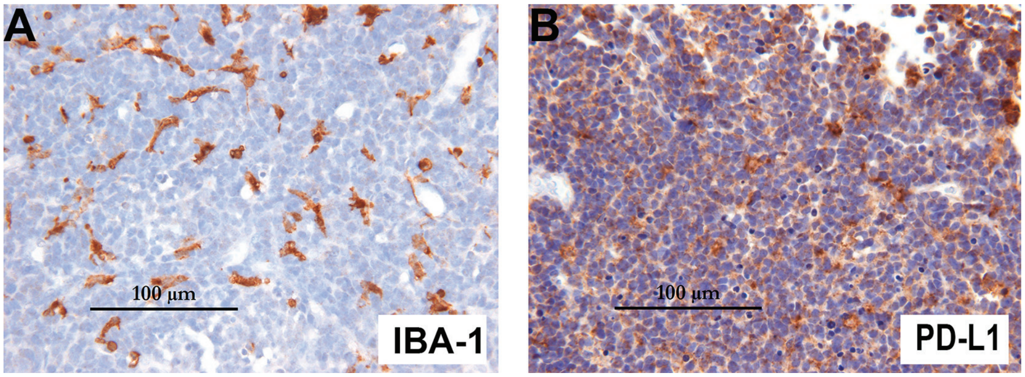

Supplementary Figure 2: WNT MB 18905 demonstrates many microglia expressing PD-L1. Immunohistochemistry of single WNT MB case stained for IBA-1 (**A**) and PD-L1 with SP-142 (**B**). There are numerous IBA-1 expressing microglial cells, and many correlate with PD-L1 positive cells.

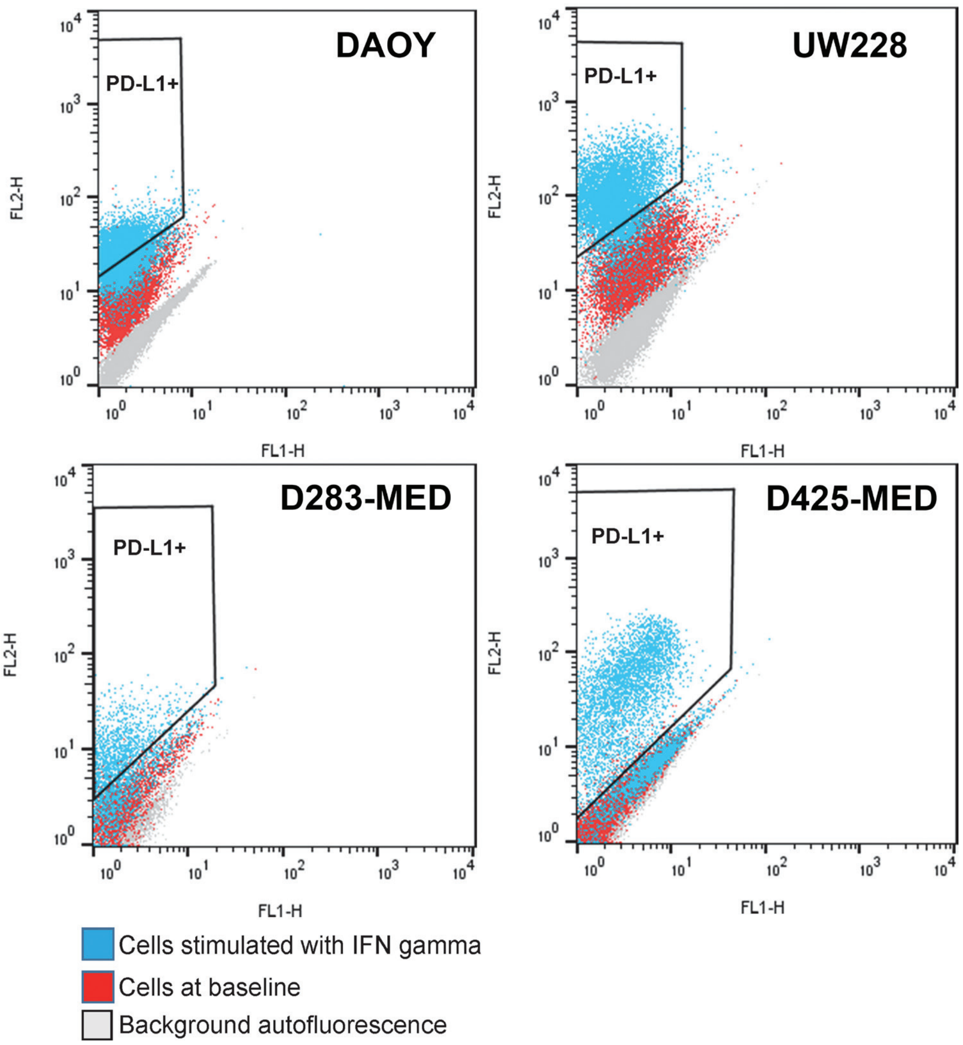

Supplementary Figure 3: Percentage of PD-L1 positive cells above baseline. Flow cytometry dot plots demonstrating different patterns in flow staining between the 4 cell lines, DAOY, UW228, D283-MED, D425-MED and the gating strategy used to gate on PD-L1+ cells (blue) above baseline (red).

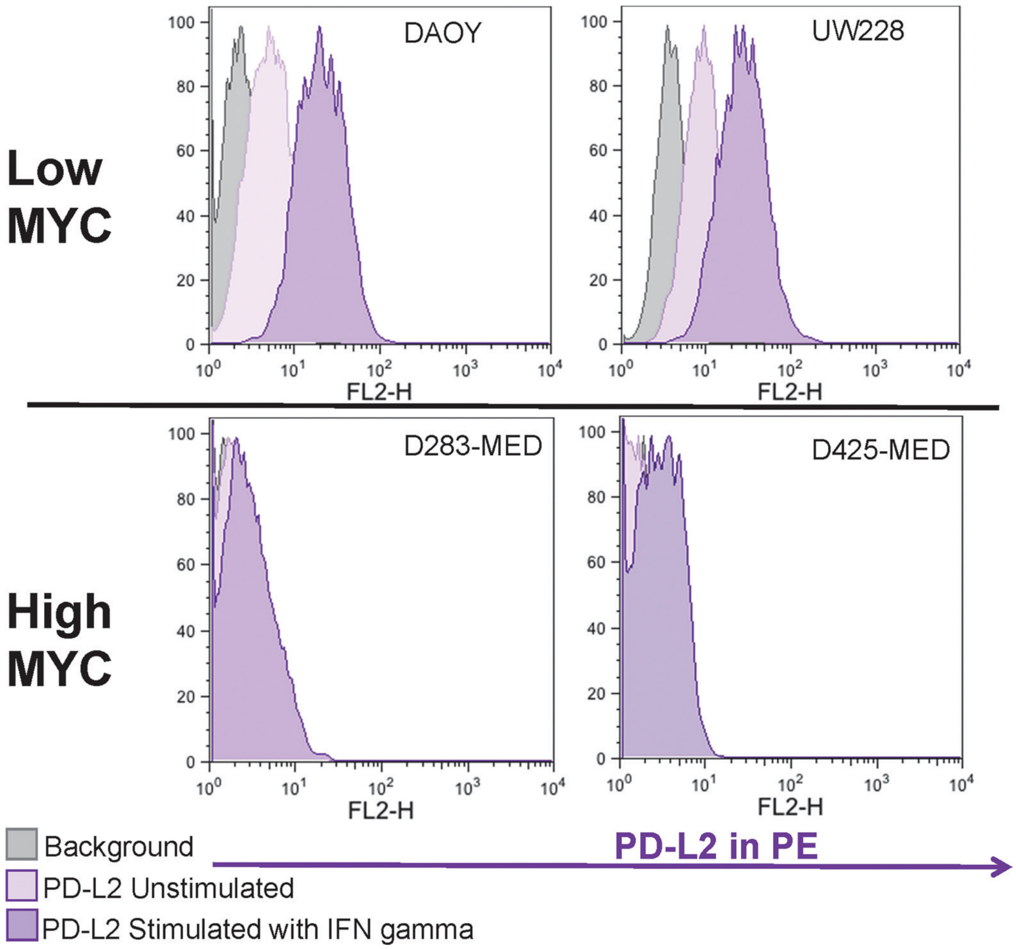

Supplementary Figure 4: PD-L2 expression has a subgroup specific pattern. PD-L2 expression in low and high MYC cell lines. Histograms show PD-L2 expression by flow cytometry using PE-conjugated MIH18 clone of PD-L2 (Ebioscience) in DAOY, UW228, D283-MED, D425-MED.

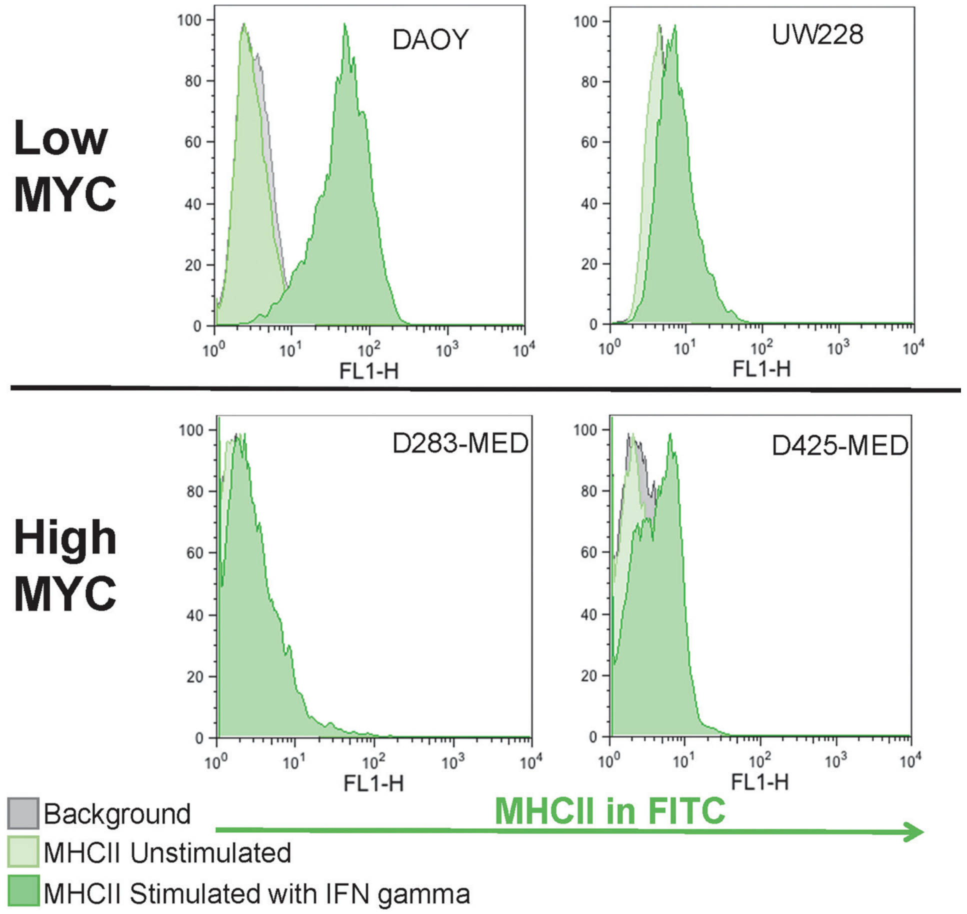

Supplementary Figure 5: MHCII expression varies independent of subgroup. MHCII expression in low and high MYC cell lines. Histograms show expression of HLA DR, DQ, and DB by flow cytometry using FITC-conjugated Tu39 clon (BD) in DAOY, UW228, D283-MED, and D425-MED.

Supplementary Table 1-1: Radiation induced PD-L1 expression as compared to IFN-γ in DAOY. See Supplementary_ Table_1-1

Supplementary Table 1-2: Radiation induced PD-L1 expression as compared to IFN-γ in UW228. See Supplementary_ Table_1-2

Supplementary Table 1-3: Radiation induced PD-L1 Expression as Compared to IFN-γ in D283-MED. See Supplementary_ Table_1-3

Supplementary Table 1-4: Radiation induced PD-L1 expression as compared to IFN-γ in D425-MED. See Supplementary_ Table_1-4
