# Supplementary material for: PD-L1 expression in medulloblastoma: an evaluation by subgroup
Source: Oncotarget. 2018 Apr 10;9(27):19177–91. doi: 10.18632/oncotarget.24951 (PMC5922386; doi:10.18632/oncotarget.24951)
Supplement: Supplementary file 2 [file oncotarget-09-19177-s002.docx]

**Supplementary Table 1-1: Radiation Induced PD-L1 Expression as Compared to IFN-γ in DAOY**

| DAOY | | | | | | |
| --- | --- | --- | --- | --- | --- | --- |
| Number of families | 3 | | | | | |
| Number of comparisons per family | 5 | | | | | |
| Alpha | 0.05 | | | | | |
|  | | | | | | |
| Dunnett's multiple comparisons test | **Mean Diff.** | **95.00% CI of diff.** | **Significant?** | **Summary** | **Adjusted P Value** | |
| 2Gy | | | | | | |
| IFN-γ vs. 2H | 21.98 | 13.79 to 30.17 | Yes | **** | 0.0001 |  |
| IFN-γ vs. 4H | 22.33 | 14.14 to 30.52 | Yes | **** | 0.0001 |  |
| IFN-γ vs. 8H | 19.93 | 11.74 to 28.12 | Yes | **** | 0.0001 |  |
| IFN-γ vs. 24H | 19.81 | 11.61 to 28 | Yes | **** | 0.0001 |  |
| IFN-γ vs. 48H | 23.1 | 14.91 to 31.3 | Yes | **** | 0.0001 |  |
| 5Gy | | | | | | |
| IFN-γ vs. 2H | 22.01 | 13.82 to 30.2 | Yes | **** | 0.0001 |  |
| IFN-γ vs. 4H | 22.04 | 13.85 to 30.23 | Yes | **** | 0.0001 |  |
| IFN-γ vs. 8H | 20.17 | 11.98 to 28.36 | Yes | **** | 0.0001 |  |
| IFN-γ vs. 24H | 18.83 | 10.64 to 27.02 | Yes | **** | 0.0001 |  |
| IFN-γ vs. 48H | 23.12 | 14.93 to 31.31 | Yes | **** | 0.0001 |  |
| 10Gy | | | | | | |
| IFN-γ vs. 2H | 21.88 | 13.68 to 30.07 | Yes | **** | 0.0001 |  |
| IFN-γ vs. 4H | 22.23 | 14.03 to 30.42 | Yes | **** | 0.0001 |  |
| IFN-γ vs. 8H | 20.31 | 12.12 to 28.5 | Yes | **** | 0.0001 |  |
| IFN-γ vs. 24H | 14.74 | 6.542 to 22.93 | Yes | *** | 0.0006 |  |
| IFN-γ vs. 48H | 22.72 | 14.52 to 30.91 | Yes | **** | 0.0001 |  |

**Supplementary Table 1-2: Radiation Induced PD-L1 Expression as Compared to IFN-γ in UW228**

| UW228 | | | | | | | |
| --- | --- | --- | --- | --- | --- | --- | --- |
| Number of families | 3 | | | | | | |
| Number of comparisons per family | 5 | | | | | | |
| Alpha | 0.05 | | | | | | |
|  | | | | | | | |
| Dunnett's multiple comparisons test | **Mean Diff.** | **95.00% CI of diff.** | **Significant?** | **Summary** | | **Adjusted P Value** | |
| 2Gy | | | | | | | |
| IFN gamma vs. 2H | -3.05 | -25.06 to 18.96 | No | ns | 0.9937 | |  |
| IFN gamma vs. 4H | 5.9 | -16.11 to 27.91 | No | ns | 0.9062 | |  |
| IFN gamma vs. 8H | 5.695 | -16.32 to 27.71 | No | ns | 0.9172 | |  |
| IFN gamma vs. 24H | 17.81 | -4.207 to 39.82 | No | ns | 0.1342 | |  |
| IFN gamma vs. 48H | 26.35 | 4.338 to 48.36 | Yes | * | 0.017 | |  |
| 5Gy | | | | | | | |
| IFN gamma vs. 2H | -0.1 | -22.11 to 21.91 | No | ns | 0.9999 | |  |
| IFN gamma vs. 4H | 4.1 | -17.91 to 26.11 | No | ns | 0.9767 | |  |
| IFN gamma vs. 8H | -3.6 | -25.61 to 18.41 | No | ns | 0.9865 | |  |
| IFN gamma vs. 24H | 17.32 | -4.697 to 39.33 | No | ns | 0.1497 | |  |
| IFN gamma vs. 48H | 26.39 | 4.381 to 48.4 | Yes | * | 0.0168 | |  |
| 10Gy | | | | | | | |
| IFN gamma vs. 2H | 5.8 | -16.21 to 27.81 | No | ns | 0.9117 | |  |
| IFN gamma vs. 4H | 6.5 | -15.51 to 28.51 | No | ns | 0.8704 | |  |
| IFN gamma vs. 8H | 5.975 | -16.04 to 27.99 | No | ns | 0.9021 | |  |
| IFN gamma vs. 24H | 18.1 | -3.917 to 40.11 | No | ns | 0.1258 | |  |
| IFN gamma vs. 48H | 25.83 | 3.822 to 47.85 | Yes | * | 0.0194 | |  |

**Supplementary Table 1-3: Radiation Induced PD-L1 Expression as Compared to IFN-γ in D283-MED**

| D283-MED | | | | | | |
| --- | --- | --- | --- | --- | --- | --- |
| Number of families | 3 | | | | | |
| Number of comparisons per family | 5 | | | | | |
| Alpha | 0.05 | | | | | |
|  | | | | | | |
| Dunnett's multiple comparisons test | **Mean Diff.** | **95.00% CI of diff.** | **Significant?** | **Summary** | **Adjusted P Value** | |
| 2Gy | | | | | | |
| IFN-γ vs. 2H | 33.22 | 15.58 to 50.86 | Yes | *** | 0.0004 |  |
| IFN-γ vs. 4H | 33.65 | 16.01 to 51.29 | Yes | *** | 0.0004 |  |
| IFN-γ vs. 8H | 29.68 | 12.03 to 47.32 | Yes | ** | 0.0012 |  |
| IFN-γ vs. 24H | 29.94 | 12.3 to 47.58 | Yes | ** | 0.0011 |  |
| IFN-γ vs. 48H | 32.48 | 14.84 to 50.12 | Yes | *** | 0.0005 |  |
| 5Gy | | | | | | |
| IFN-γ vs. 2H | 33.56 | 15.91 to 51.2 | Yes | *** | 0.0004 |  |
| IFN-γ vs. 4H | 32.11 | 14.46 to 49.75 | Yes | *** | 0.0006 |  |
| IFN-γ vs. 8H | 24.31 | 6.669 to 41.95 | Yes | ** | 0.0062 |  |
| IFN-γ vs. 24H | 29.61 | 11.96 to 47.25 | Yes | ** | 0.0012 |  |
| IFN-γ vs. 48H | 32.11 | 14.46 to 49.75 | Yes | *** | 0.0006 |  |
| 10Gy | | | | | | |
| IFN-γ vs. 2H | 28.85 | 11.21 to 46.49 | Yes | ** | 0.0015 |  |
| IFN-γ vs. 4H | 33.73 | 16.09 to 51.37 | Yes | *** | 0.0004 |  |
| IFN-γ vs. 8H | 6.35 | -11.29 to 23.99 | No | ns | 0.7659 |  |
| IFN-γ vs. 24H | 28.07 | 10.42 to 45.71 | Yes | ** | 0.0019 |  |
| IFN-γ vs. 48H | 33 | 15.36 to 50.64 | Yes | *** | 0.0004 |  |

**Supplementary Table 1-4: Radiation Induced PD-L1 Expression as Compared to IFN-γ in D425-MED**

| D425-MED | | | | | | |
| --- | --- | --- | --- | --- | --- | --- |
| Number of families | 3 | | | | | |
| Number of comparisons per family | 5 | | | | | |
| Alpha | 0.05 | | | | | |
|  | | | | | | |
| Dunnett's multiple comparisons test | **Mean Diff.** | **95.00% CI of diff.** | **Significant?** | **Summary** | **Adjusted P Value** | |
| 2Gy | | | | | | |
| IFN-γ vs. 2H | 37.84 | 22.05 to 53.63 | Yes | **** | 0.0001 |  |
| IFN-γ vs. 4H | 37.59 | 21.79 to 53.38 | Yes | **** | 0.0001 |  |
| IFN-γ vs. 8H | 36.91 | 21.11 to 52.7 | Yes | **** | 0.0001 |  |
| IFN-γ vs. 24H | 37.32 | 21.52 to 53.11 | Yes | **** | 0.0001 |  |
| IFN-γ vs. 48H | 37.11 | 21.31 to 52.9 | Yes | **** | 0.0001 |  |
| 5Gy | | | | | | |
| IFN-γ vs. 2H | 37.8 | 22 to 53.59 | Yes | **** | 0.0001 |  |
| IFN-γ vs. 4H | 37.78 | 21.98 to 53.57 | Yes | **** | 0.0001 |  |
| IFN-γ vs. 8H | 36.94 | 21.15 to 52.73 | Yes | **** | 0.0001 |  |
| IFN-γ vs. 24H | 36.73 | 20.94 to 52.52 | Yes | **** | 0.0001 |  |
| IFN-γ vs. 48H | 36.54 | 20.74 to 52.33 | Yes | **** | 0.0001 |  |
| 10Gy | | | | | | |
| IFN-γ vs. 2H | 37.88 | 22.09 to 53.67 | Yes | **** | 0.0001 |  |
| IFN-γ vs. 4H | 37.77 | 21.98 to 53.56 | Yes | **** | 0.0001 |  |
| IFN-γ vs. 8H | 36.65 | 20.85 to 52.44 | Yes | **** | 0.0001 |  |
| IFN-γ vs. 24H | 36.98 | 21.18 to 52.77 | Yes | **** | 0.0001 |  |
| IFN-γ vs. 48H | 35.58 | 19.79 to 51.37 | Yes | **** | 0.0001 |  |
